# The Potential Use of Digital Twin Technology for Advancing CAR-T Cell Therapy

**DOI:** 10.3390/cimb47050321

**Published:** 2025-04-30

**Authors:** Sara Sadat Aghamiri, Rada Amin

**Affiliations:** 1 Center for Brain, Biology and Behavior, University of Nebraska, Lincoln, NE 68503, USA; 2 Department of Biochemistry, University of Nebraska, Lincoln, NE 68503, USA

**Keywords:** CAR-T cells, digital twins, immunotherapy, cancer, tumor microenvironment, tumor antigens

## Abstract

CAR-T cell therapy is a personalized immunotherapy that has shown promising results in treating hematologic cancers. However, its therapeutic efficacy in solid cancers is often limited by tumor evasion mechanisms, resistance pathways, and an immunosuppressive tumor microenvironment. These challenges highlight the need for advanced predictive models to better capture the intricate interactions between CAR-T cells and tumors to enhance their potential. Digital Twins represent a transformative approach for optimizing CAR-T cell therapy by providing a virtual representation of the therapy-tumor trajectory using high-dimensional patient data. In this review, we first define Digital Twins and outline the fundamental steps in their development. We then explore the critical parameters required for designing CAR-T-specific Digital Twins. We examine published case studies demonstrating a few applications of Digital Twins in addressing key challenges in CAR-T cell therapy, including their impact on clinical trials and manufacturing processes. Finally, we discuss the limitations associated with integrating Digital Twins into CAR-T therapy. As Digital Twin technology continues to evolve, the potential to enhance CAR-T therapy through precision modeling and real-time adaptation could redefine the landscape of personalized cancer treatment.

## 1. Introduction

Chimeric Antigen Receptor T (CAR-T) cell therapy has demonstrated encouraging outcomes in treating refractory hematologic cancers [1]. Since 2017, the U.S. Food and Drug Administration (FDA) has approved CAR-T cell therapies for B-cell acute lymphoblastic leukemia, follicular lymphoma, diffuse large B-cell lymphoma, mantle cell lymphoma, and multiple myeloma, marking a significant milestone in personalized cancer treatment [2]. This innovative therapy involves the use of the patient’s T cells, which are genetically modified to express synthetic CARs that can specifically recognize and bind to antigens on the surface of cancer cells. Once infused back into the patient, CAR-T cells actively target and destroy malignant cells by binding to these antigens, triggering a potent immune response. Despite significant results in hematologic cancers, CAR-T therapy has not shown similar efficacy in broader applications, especially in solid tumors. The main obstacles include variability in tumor antigen expression, an immunosuppressive tumor microenvironment (TME), reduced T cell penetration to the tumor site, and treatment-related toxicities such as cytokine release syndrome (CRS) and neurotoxicity [3]. Moreover, T-cell exhaustion and loss of functionality over time can also limit the durability of the therapeutic response [4]. These challenges necessitate advanced solutions that integrate the system as a whole to enhance the therapeutic potential of CAR-T cells despite the tumor ecosystem.

To overcome these limitations, computational approaches have become increasingly popular for addressing the complexities of CAR-T cell therapy. Computational tools that integrate molecular, cellular, and clinical datasets are invaluable for modeling CAR-T cell behavior under diverse conditions [5,6]. For instance, computational modeling can predict patient responses based on CAR T-cell dynamics [7] or identify optimal tumor-associated antigen binders for CAR constructs [8]. Moreover, to address the dynamic crosstalk between CAR T-cells and the tumor ecosystem, real-time data-driven approaches are essential. Unlike static models, which provide a fixed snapshot of CAR T-cell behavior without accounting for temporal or environmental variations, dynamic models simulate their real-time interactions with the TME, capturing adaptive responses to microenvironmental pressures and enabling more accurate predictions of treatment outcomes. This type of model will not only provide virtual tracking of CAR T-cell efficacy but also facilitate treatment adjustments to minimize side effects accordingly [9].

Digital twin technology represents a cutting-edge solution with the potential to advance CAR-T therapy by bridging the gap between computational modeling and clinical application [10]. A digital twin (DT) is a virtual replica of a real-world system that is continuously updated with data to mirror and predict the behavior of its physical counterpart [11]. The concept of the DT originated in engineering; however, in recent years, its applications have expanded to healthcare and precision medicine [9,12]. Various DT pilots have been developed by leading research centers and universities to address the key challenges in solid cancers. These DT models integrate retrospective multimodal data and patient-specific tumor profiles to guide treatment interventions [13]. Beyond prediction capability, these virtual models can evolve in real-time, using patient monitoring data to improve simulations and direct individualized therapeutic interventions. This dynamic capability can be particularly useful for the management of adverse events associated with CAR-T cells based on their predictive insights. DTs can not only transcend the present constraints of CAR-T therapy but also allow for a more accurate, flexible, and patient-centered approach to immunotherapy by providing researchers and physicians with an advanced tool for decision-making.

In this review, we first define DTs and outline the key steps involved in their development and application. We then categorize the essential factors to be considered when designing CAR-DTs. In addition, we highlight several case studies demonstrating how DTs have been applied to address critical challenges in CAR-T cell therapy, including those related to kinetics, clinical trials, and manufacturing processes. Finally, we discuss the challenges of implementing DTs in CAR-T therapy and identify the key barriers that must be overcome for further advancement.

## 2. Digital Twin Technology

### 2.1. General Definition

A DT is defined as a digitalized replica of a physical system or environment that mirrors its real-world counterpart and predicts its behavior at future time points. The concept of DTs initially originated in the aerospace and manufacturing industries. Engineers have created virtual models of complex systems, such as aircraft, jet engines, and industrial machinery, to monitor their performance, predict failures, and optimize maintenance [14]. Over time, as computational power, artificial intelligence (AI), and data analytics have advanced, the application of DTs has expanded beyond engineering to industries such as smart cities, transportation, and, eventually, healthcare [15]. Moreover, the evolution from a digital model to a digital shadow to a DT reflects the increasing integration between the digital and physical worlds. A digital model is a standalone virtual representation of a system built using data, rules, or simulations but is not connected to any physical counterpart. A digital shadow adds a one-way link: data from the physical system automatically updates the digital model, enabling real-time monitoring but not controlling. Finally, the DT forms a two-way, real-time connection. The digital system not only receives data from the physical world but can also influence or control it, allowing continuous synchronization, prediction, optimization, and decision-making across both domains [16,17].

In precision medicine, the physical asset that DTs replicate is the individual patient, along with organs, tissues, and physiological processes that are continuously updated based on historical and real-time patient data. The growing availability of patient-specific data, including imaging, next-generation sequencing, biopsies, digitized immunohistochemistry, and electronic health records, offers a data foundation for developing patient-centric DTs in medicine [18,19]. For instance, a multicenter team is developing the first single-cell computer model of child development and cancer condition, called virtual child, to be able to simulate unlimited virtual trials to accelerate pediatric cancer treatments [20]. Similarly, DTs are being developed at the tissue scale, with a focus on specific organs and their disease associations, including heart dysfunction [21], neurodegenerative brain diseases [22,23], and respiratory lung diseases [24]. DTs are increasingly being proposed and adopted across diverse medical fields to advance personalized diagnostics treatment planning and improve disease monitoring. In cardiology, DTs model heart dynamics to optimize diagnosis, treatment, surgical planning, and cardiac monitoring [25]. In neurology, DTs help track and predict neurodegenerative disease progression, such as dementia [26], Alzheimer’s disease [27], Parkinson’s disease [28], and multiple sclerosis [29]. Applications also include orthopedics for joint modeling [30], diabetology for personalized glucose control [31], and nephrology [32]. The future of DTs in healthcare is set to advance precision medicine with primary applications in diagnosis, therapeutic strategies, monitoring, and research and development. Their use spans various fields of medicine, including hospital management, emergency care, medical device development, biomarker and drug discovery, biomanufacturing, surgical planning, in silico clinical trials, wellness monitoring, and personalized medicine [11]. The wide range of applications highlights the growing significance of DTs and their transformative potential in the field of healthcare.

One of the most promising applications of DTs in precision medicine is in oncology [12], where cancer digital twins are emerging as powerful tools for modeling tumor evolution, predicting therapy resistance, and developing personalized biomarker panels for non-invasive monitoring [33]. Given the complexity of cancer, a sophisticated approach is essential to fully understand the challenges it poses to therapy. In this context, DTs are expected to function as digital duplicates of the patient’s tumor, combining molecular profiles, cellular dynamics, genetic variations, spatial tumor organization, and therapeutic interventions to produce a customized model [13]. In addition, the accumulation of individual DTs per patient over time can be leveraged to generate a virtual population-wide cohort, adding more granularity to the development of treatment guidelines [34]. Clinicians may be able to make more individualized treatment decisions if DT models are improved and incorporated into the clinical routine workflow. Before directly testing novel therapies on patients, these models can assist clinicians in testing various regimens, modifying dosages, or investigating alternate treatment options, greatly lowering risks and enhancing efficacy [35].

### 2.2. Development of DTs

**Figure 1:**
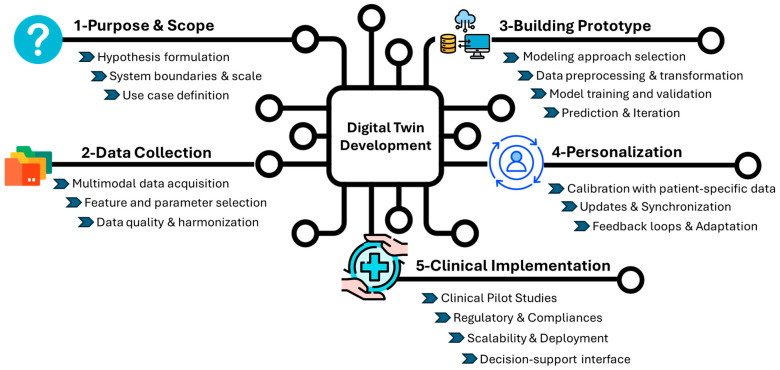
General development of the Digital Twin. The figure outlines the key stages in the development and clinical integration of DT for healthcare applications. The process begins with the purpose and scope (1), where a hypothesis is defined, system boundaries and scale are determined, and use cases are identified. In data collection (2), multimodal data (clinical, omics, and imaging) are gathered, key parameters are selected, and datasets are harmonized. Building the prototype (3) involves choosing a computational approach, preprocessing data, training, validating the model, generating predictions, and continuously refining the system through iterative improvements. The personalization stage (4) includes calibrating the model with individual patient data, synchronizing updates from longitudinal records, and adapting to feedback loops. Finally, clinical implementation (5) could begin with pilot testing and real-world validation, enabling scalable and regulatory-compliant deployment and supporting clinical decision-making through intuitive user interfaces.

Digital twins are dynamic, bidirectional models that evolved alongside their physical counterparts. This technology has the potential to address several key points: (i) identifying potential failures before they occur to enhance safety, (ii) optimizing operations through real-time adjustments to ensure performance, and (iii) reducing resource consumption and operational costs to ultimately increase efficiency. To achieve an “ideal” DT, the design requires a multi-step approach to reflect the complexity of the system (Figure 1).

#### 2.2.1. Define the Hypothesis and Scope of the Model

The first step is to outline the specific clinical or biological question that the model aims to address and the scale at which the model will operate. This could range from molecular interactions, such as signaling pathways and genetic alterations, to cellular dynamics and even extend to patient treatment. Alternatively, the model may need to integrate both levels for a more holistic view. In the context of CAR-T cells, one key question is how components of the TME, especially in solid cancers, such as cytokine gradients, immunosuppressive cells, and metabolic constraints, influence CAR-T cell persistence and therapeutic efficacy. At the clinical scale, DTs can help address the complex balance between efficacy and toxicity, predict early toxicity responses, optimize dose selection, and design trials.

The single-scale model might focus on a specific aspect, like T cell signaling, while a multi-scale model would incorporate both cellular and molecular interactions, as well as systemic factors such as immune response and tumor progression. Multi-scale modeling offers a more comprehensive understanding of how various factors interact at different levels, which is essential for predicting therapy outcomes [36].

The scale at which the model operates is largely dictated by the availability of data and the clinical or research objectives. For instance, if single-cell sequencing of CAR-T cell patient data is available, the model could operate at a highly granular level, capturing interactions at the molecular or even sub-cellular scale [37]. Conversely, if clinical trial data or patient outcomes are the primary sources of information, the model may need to integrate larger-scale patient-level data, focusing on treatment efficacy, relapse patterns, and therapy adjustments over time [38]. Although some models can be built despite limited data, this may impact the robustness of the model prediction [39].

Identifying and listing all components and parameters that should be integrated to create a comprehensive and accurate simulation is essential. For instance, the Stanford University group created an adaptive cancer patient digital twin (CPDT) that leverages retrospective cohorts by incorporating demographic, imaging, genomic, and histopathology RNA-sequencing data. This data will then be integrated into the CPDT for further refinement. The goal of the CPDT is to monitor treatment response, predict resistance, and optimize treatment reassignment in lung cancer [13].

A diverse array of multimodal data can be leveraged to effectively model CAR-T cell therapy using DTs for better prediction. This includes patient-specific clinical data (e.g., baseline blood counts, inflammatory markers, and cytokine profiles), genomic and transcriptomic information (from both tumor and T cells), CAR-T cell product characteristics (such as transduction efficiency, phenotypic composition, and exhaustion markers), imaging data (for tracking tumor burden, toxicity, and CAR-T trafficking), and dynamic response data post-infusion (e.g., serial cytokine levels, CAR-T cell expansion curves, and toxicity grading) [40].

#### 2.2.2. Construct a Baseline Template Model

The next foundational step is the construction of a baseline template model. The model should serve as a generic model that integrates key biological and clinical knowledge and provides a starting point for more detailed and patient-specific simulations. The baseline model acts as a standard to which all future refinements, updates, and patient-specific variations will be compared. Therefore, it is important to make the framework modular and adaptable, allowing for the integration of patient-specific data as they become available [41].

For the initial template, the DT will be constructed using historical patient data that reflect both past clinical outcomes and established knowledge, thereby serving as a baseline model. The growing number of completed and ongoing clinical trials of CAR-T therapy can be a valuable foundation for building clinical models that capture the diversity of real-world patient responses [38].

Computational approaches must be tailored to each data type to effectively handle and integrate diverse sources of information [42]. For clinical data from human patients treated with CAR-T cells, approaches such as machine learning, statistical modeling, and pattern recognition algorithms can be employed to identify hidden relationships and predict patient-specific outcomes [43]. Experimental system data, including high-throughput omics or single-cell sequencing, may require advanced computational techniques like network analysis, integrative multi-omics approaches, or deep learning models to uncover complex interactions at the molecular or cellular level. In all cases, the computational approaches should be selected based on their ability to capture the key features of the system being modeled, as well as their capacity to integrate and process large-scale data from different biological layers (genomic, transcriptomic, proteomic, etc.) [44].

To ensure the accuracy and reliability of the model predictions, it is essential to use known experimental or clinical data to parameterize and validate the first version. By incorporating data from these different sources, the model can be rigorously tested and fine-tuned to quantify the uncertainty across various scenarios. This approach ensures that the model is grounded in empirical evidence, accounting for the complexity and heterogeneity inherent to biological systems. Furthermore, validation against multiple data types allows for a more comprehensive understanding of the model’s behavior and enhances its predictive capabilities, ensuring its robustness and relevance to clinical and experimental applications.

#### 2.2.3. Advancing Personalized Solutions Through Patient-Centric Models

The generic model should be personalized for individual patients to allow for broader clinical application. This phase transforms the generic baseline model into a dynamic, patient-specific simulation tool capable of predicting treatment outcomes and guiding clinical decision-making. Personalization ensures that the model accurately reflects the unique biological, genetic, and clinical characteristics of each patient, allowing for more precise and tailored therapeutic interventions to be developed. Initial personalization can be based on retrospective and prospective clinical trial data to validate accuracy [13]. Personalization begins by integrating observation patient-specific data into the DT model as inputs, which can include, for example, imaging scans taken at different time points, providing detailed information on tumor growth and metastasis. The model can generate outputs that track tumor progression or regression, helping to predict the effectiveness of current treatments [45]. For example, DTs can offer a personalized approach by integrating patient-specific variables that distinguish between responders, non-responders, and those who experience relapse. In this model, personalization could involve incorporating several parameters, such as initial response, CAR-T cell kinetics, tumor proliferation, tumor burden, and toxicity assessment, to understand the heterogeneity in patient responses and predict long-term outcomes. This level of personalization not only helps optimize treatment strategies for each patient but also provides predictive insights into why certain patients fail to respond or relapse after an initial response [46].

Integrating patient-specific data into the model must be calibrated to adapt to the unique biological environment of each individual. This involves adjusting the parameters within the model by testing significant input/output relationships to align with the patient’s specific data. Calibration may include fine-tuning the model’s assumptions regarding tumor dynamics, immune system interactions, or treatment response based on the patient’s status and previous treatment outcomes. For example, liquid biopsy provides a minimally invasive approach to monitor patient response to CAR-T cell treatment through real-time insights into circulating tumor DNA, immune cell profiles and therapy-induced molecular changes [47,48]. Moreover, radiological imaging techniques, such as positron emission tomography/computed tomography and magnetic resonance imaging (MRI), further complement this approach by tracking toxicity and therapy response [49]. Interestingly, Wu et al. developed a DT framework using longitudinal retrospective MRI data of triple-negative breast cancer patients treated with neoadjuvant chemotherapy. The model can predict the treatment schedule for individual patients to improve clinical outcomes, thereby leading to personalized treatment strategies [50].

The frequency of data updates plays a crucial role in maintaining the model’s relevance and accuracy; regular updates, whether through periodic tests, imaging scans, or monitoring of biomarkers, are essential for synchronizing the two entities, capturing changes in the patient’s condition, and quantifying uncertainty [33].

This ongoing learning process is crucial for maintaining the model’s predictive capability, as it must reflect the latest biological and clinical developments in the patient’s condition.

#### 2.2.4. DT Models—For Clinical Routine Implementations

The bidirectional communication loop between the virtual model and the physical patient differentiates DTs from conventional computational models. In clinical routine pipelines, this creates a powerful feedback system in which the DT evolves alongside the patient’s disease trajectory. DTs operate within a dynamic, high-dimensional, and context-aware data environment to reflect the most current and granular patient data [51].

When integrated into clinical routine pipelines, DTs can transform reactive care into proactive, data-driven decision-making. Several clinical trials have begun integrating DT frameworks into their design, targeting conditions such as type 2 diabetes (NCT05181449), musculoskeletal injuries (NCT04849923), insulin delivery, and meal prediction algorithms (NCT04203823), as well as assessing triglyceride and glucose responses for tailored dietary recommendations (NCT05313594).

For the development of a clinical trial involving CAR-T cells, DTs can replace traditional control arms by simulating patient-specific disease progression and treatment responses. Based on the framework proposed by Thangaraj et al. [52], DTs can simulate randomized clinical trial (RCT) outcomes in real-world populations by conditioning generative models using electronic health record data. Therefore, this approach can support the real-time in silico evaluation of CAR-T cell therapy efficacy and safety without the ethical concerns associated with withholding potentially beneficial treatments. Furthermore, it can also facilitate the direct translation of RCT findings into clinical practice, enhance trial generalizability, and support the development of personalized CAR-T cell therapy strategies [52]. These examples demonstrate the growing potential of DTs to enhance clinical trial design by simulating the control arms and supporting adaptive decision-making throughout treatment.

Given the need for a multi-scale data approach to build DTs, adherence to the FAIR (Findable, Accessible, Interoperable, and Reusable) data principles is fundamental to ensure transparency, reproducibility, and interoperability across systems and institutions. FAIR-compliant data management not only improves model traceability and validation but also fosters collaboration, regulatory alignment, and scalability [53]. These principles are particularly critical when DTs are used in regulated clinical environments, where trust, data provenance, and accountability are essential. Clinical approval requires adherence to rules like FDA or EMA (European Medicines Agency) standards, which entail thorough validation to guarantee safety, dependability, and clinical value. Transparency during regulatory evaluations requires thorough documentation of the model creation process, including the input data, algorithms, and validation techniques. Data security and patient privacy are also crucial since handling sensitive data necessitates compliance with regulations like HIPAA (Health Insurance Portability and Accountability Act) or GDPR (General Data Protection Regulation) [54]. Notably, government bodies such as the FDA, NSF, NIH, and European Commission are increasingly investing in DT technologies, recognizing their transformative potential for next-generation biomedical solutions [55]. These efforts reflect the growing momentum toward adopting DTs to advance precision medicine, clinical decision-making, and patient care.

To enable widespread adoption, additional steps must include ensuring scalability, a robust infrastructure, workforce training, and standardized integration across diverse clinical settings. A well-designed, FAIR-aligned decision-support interface must be developed to translate complex model outputs into intuitive and actionable insights for clinicians, positioning DTs as copilots that enhance but do not override clinical judgment. Close collaboration between development teams and regulatory agencies is essential to align with all the necessary standards for clinical implementation in precision medicine [35,56].

## 3. Where Does CAR-T Cell Therapy Stand in the DT Context?

**Figure 2:**
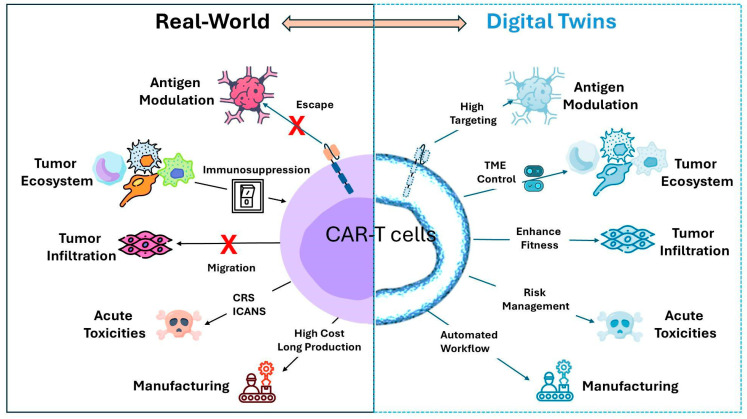
Bidirectional connection between the Real-World and Digital Twins for CAR-T cell therapy. The real-world counterparts include the main factors that reduce CAR-T cell efficacy. The DTs box represents the main solutions that DTs can tackle to enhance and improve CAR therapy. CRS, cytokine release syndrome; ICANS, Immune Effector Cell-Associated Neurotoxicity Syndrome; and TME, tumor microenvironment.

To address this question, it is essential to first define the physical system that the DTs should replicate. In the context of CAR-T cell therapy, the DT must model the tripartite interactions between CAR-T cells, tumor cells, and the TME, including critical elements such as immune checkpoint molecules, cytokine profiles, and potential resistance mechanisms that may influence therapy outcomes. To better understand and predict the success of CAR-T therapy, we summarized the most critical components, such as antigen heterogeneity, TME-mediated immune suppression, tissue trafficking, acute toxicities, and manufacturing, which can significantly compromise CAR-T cell efficacy (Figure 2).

### 3.1. Antigen Heterogeneity

The optimal killing of tumors by CAR-T cells relies on precise molecular interactions between engineered TCR cells and tumor antigens. However, target antigen heterogeneity, tumor escape, and tumor-induced immune suppression present significant barriers to CAR-T cell therapy efficacy.

Antigen expression exhibits significant heterogeneity, varying not only between different tumor sites within the same patient but also within the tumor itself due to its hierarchical organization. In addition, expression patterns can shift between primary and recurrent tumors, as well as before and after treatment, adding challenges for antigen selection [57]. While early CAR-T cells were limited to one surface antigen, novel strategies have been developed to address antigen heterogeneity by combining multiple chimeric receptors for different targets or designing a biotin-binding immune receptor as a universal CAR to bind biotinylated cancer cells [58].

Moreover, tumor-specific antigens are exclusively expressed by cancer cells [59]. CAR-T cells primarily target tumor-associated antigens, which are highly expressed in cancer cells but expressed at low levels in normal tissues, increasing the risk of on-target-off-tumor toxicities and potential damage to healthy cells [60].

Another limitation of CAR therapy is further exacerbated when the antigen is downregulated, limiting the interaction between the target and CAR-T cells. For example, treatment failure has been observed with CD19-CAR therapy; despite achieving remission rates of 70–90%, many patients eventually relapse due to antigen loss or downregulation [61]. Similarly, relapsed or refractory patients with pre-B-cell acute lymphoblastic leukemia treated with CD22-CAR-T therapy after CD19-CAR failure often experience relapse due to a reduction in CD22 expression despite the persistence of CD22-CAR cells [62].

Approximately 30 to 70% of patients experience antigen loss following CAR-T therapy, a process driven by several complex mechanisms, including genetic instability [63], epigenetic programming [64], antigen shedding [65], and clonal selection [66]. Time-series single-cell data could be integrated within the DT framework, as previously published by Li et al., to model genetic and molecular changes across diverse cell types, providing a deeper understanding of antigen changes during therapy response [67]. To address antigenic changes and clonal heterogeneity, DTs can integrate longitudinal data on tumor antigen expression, single-cell transcriptomics, and spatial heterogeneity of CAR-T cells in co-culture with tumor cells. This dynamic model can simulate tumor evolution and suggest potential strategies for overcoming antigen escape, such as identifying more stable antigens or designing CAR-T cells to target alternative or multiple antigens. By incorporating data on co-expressed antigens (e.g., CD22 and CD123), DT can also assess the potential benefits of dual-targeting strategies or universal CAR platforms tailored to the individual patient’s tumor profile.

CAR-T cells require high antigen expression for effective targeting; however, continuous exposure to tumor antigens drives progressive CAR-T cell exhaustion, reducing their effector functions and proliferation [4,68]. Interestingly, tumor cells actively transfer antigens to CAR-T cells through a process called trogocytosis, which reduces antigen expression and impairs CAR-T function. This weakens tumor targeting by CAR-T cells and triggers fratricide, in which CAR-T cells attack each other, leading to exhaustion and diminished therapeutic efficacy. This phenomenon has been observed in leukemia in vivo models of CD28- and 4-1BB-based CARs [69].

Overall, DTs designed to guide or optimize CAR-T cell therapies should incorporate the critical role of antigen characteristics, namely accessibility, density, heterogeneity, and specificity, which significantly influence CAR-T cell engagement, synapse formation, and subsequent cytotoxicity toward target cells. These antigen-related parameters are not static; they can evolve due to tumor adaptation, therapy-induced modulation, and microenvironmental influences. To achieve this, it is vital to integrate high-resolution imaging and multi-scale omics data that can characterize and monitor antigen expression over time and across tumor regions [66]. Techniques such as flow cytometry, immunohistochemistry, and next-generation sequencing can provide quantitative single-cell data on the expression of surface antigens. Additionally, emerging tools like digital spatial profiling (DSP) and multiplexed imaging allow for the in situ analysis of antigen co-expression patterns within the TME, offering insights into spatial heterogeneity and potential immune escape mechanisms. Ultimately, antigen-centric modeling is a key pillar in constructing clinically actionable DTs to dynamically model tumor antigen changes after CAR-T cell therapy.

### 3.2. Tumor Microenvironment

Developing an accurate digital twin of the TME requires a deep understanding of its cellular and molecular complexity. The TME’s immunosuppressive landscape, including inhibitory cells, cytokine signaling, and physical barriers, critically impacts CAR-T cell efficacy. A well-designed DT must capture these dynamic interactions to optimize therapy and overcome resistance mechanisms.

One complex aspect of cancer is the critical organization of the niche in which the cancer cells reside. The tumor microenvironment consists of well-organized niches that support tumor survival, resistance, and escape through several cellular and molecular mechanisms [70]. At the cellular organization, a wide range of cells infiltrate the TME with immunosuppressive functions, which have been strongly correlated with CAR-T efficacy and clinical outcomes [71].

Among these immunosuppressive cells, the TME exhibits infiltration of several key immune cell populations, including regulatory T cells (Tregs), tumor-associated macrophages (TAMs), and myeloid-derived suppressor cells (MDSCs). These cells share functional similarities in suppressing anti-tumoral immunity and also work in concert to ultimately contribute to tumor progression and resistance to immunotherapy. For instance, these cells secrete several immunosuppressive cytokines, such as transforming growth factor-beta (TGF-β) and interleukin-10 (IL-10), which hinder the effector function of T cells [72]. TAMs and MDSCs share immunosuppressive mechanisms, including the production of reactive oxygen species, arginase-1, and inducible nitric oxide synthase, which collectively deplete essential nutrients, such as L-arginine, tryptophan, and cysteine, from the TME. This metabolic deprivation creates a hostile landscape for T cell function, leading to nutrient starvation that impairs T cell proliferation, activation, and effector responses [73]. Di Filippo et al. developed a metabolic DT framework called single-cell flux balance analysis, which was designed to create digital metabolic twins by integrating single-cell RNA-seq data into population-based models. The authors aimed to characterize single-cell metabolic changes along with the unsupervised identification of metabolic subpopulations, offering a valuable complement to patient-derived cellular models [74]. A similar framework, adapted to CAR-T cells, could be used to investigate metabolic fluctuations and predict novel metabolic targets that hinder CAR-T cell functionality.

In addition, crosstalk between these cells mutually enhances their expansion and reinforces their immunosuppressive functions. For instance, Tregs suppress cytotoxic CD8+ T lymphocytes, thereby indirectly lifting the inhibitory effects of CD8-derived IFN-γ on the development and function of M2-like macrophages [75]. Moreover, MDSCs promote Treg differentiation through direct cell-cell interactions or by secreting immunosuppressive cytokines, such as IL-10 and TGF-β. Conversely, Tregs further enhance the immunosuppressive function of MDSCs by upregulating inhibitory receptors and stimulating IL-10 production, creating a reinforcing loop that strengthens immune suppression in the TME [76].

Platforms such as organoids, lab-on-chip platforms, and other customizable microphysiological systems are increasingly used to replicate the structural and functional complexity of the TME [77]. For instance, Logun et al. demonstrated that glioblastoma organoids generated in parallel from patients undergoing CAR-T cell therapy exhibited a comparable temporal pattern in cytokine release, cytotoxicity, CAR-T cell activation, and tumor cell lysis to that observed in the corresponding clinical trial participants [78]. This suggests that organoids can be a suitable real-time monitoring system for investigating the crosstalk between the TME and CAR-T cells. Depending on the research goal, 2D and 3D systems can be tailored to incorporate CAR-T cells with various stromal and immune components, tumor subtypes, and even perfusion to mimic vascular flow [79].

At the molecular level, immunosuppressive cells sequester vital cytokines like IL-2 due to the expression of high-affinity receptors on Treg, depriving effector T cells of necessary survival and expansion signals in addition to their exhaustion [80,81]. Adding IL-2 is not beneficial for CAR-T cells, as it primarily promotes the expansion of Tregs within the TME, further suppressing effector T cell activity [82]. Some strategies aim to overcome IL-2 deprivation by co-expressing mutated IL-2 with CAR-T cells [83,84], enhancing the interaction between the modified IL-2 and the engineered receptor rather than relying on endogenous IL-2. However, additional cytokines, including IL-7, IL-15, and IL-21, can influence T-cell homeostasis and are being tested in multiple clinical trials [85]. While these approaches based on IL-2 have shown significant improvements in CAR-T cell anti-tumor activity in vivo, challenges remain, including identifying the optimal strategy for cytokine mutation, selecting the most effective cytokines, ensuring long-term efficacy, and mitigating potential side effects in clinical applications [85].

Immune checkpoint inhibitors play a critical role in the exhaustion and immunosuppressive function of the TME. CAR-T cells often express PD-1, which, upon interaction with PD-L1 on tumor cells and MDSCs, triggers inhibitory pathways that further impair their function [86]. Several studies have attempted to inhibit PD-1 expression on CAR-T cells with opposite results. For instance, Wei et al. generated a CAR-T with a permanent KO of PD-1 expression. Although in vitro studies have shown effective CAR-T cell function, in vivo studies have shown a limitation of CD19-CAR-T cells to proliferate and differentiate into memory, thereby limiting their anti-tumoral properties in the long term [87]. In contrast, a study by Ouyang et al. demonstrated that knocking down PD-1 in BCMA-CAR-T cells enhanced memory differentiation and reduced exhaustion, even after repeated antigen stimulation in vitro, resulting in optimal anti-tumor activity in vivo. Furthermore, a Phase I clinical trial showed an 86% response rate (of 6/7 patients with refractory/relapsed multiple myeloma) with mild-to-moderate CRS [86]. Audreu-Saumell et al. developed a preclinical model to assess how CAR affinity affects PD-1/PD-L1 inhibition. They found that low-affinity (LA) CAR-T cells were more susceptible to PD-1/PD-L1 inhibition than high-affinity (HA) CAR-T cells. They disrupted PD-1 and improved LA CAR-T cell function but had no effect on HA CAR-T cells, especially with CD28 and ICOS co-stimulation. 4-1BB co-stimulation conferred resistance to inhibition, regardless of CAR affinity. These studies highlight that the CAR category and affinity for tumor antigens determine sensitivity to PD-L1 inhibition [88].

As the tumor progresses, the TME becomes stiffer due to the overproduction of matrix molecules, including collagen, fibronectin, and heparan sulfate proteoglycans. This increased rigidity limits the ability of CAR-T cells to penetrate the tumor, which can be limited by the enzymatic action of matrix metalloproteinases (MMP) [89] and heparanase [90] to degrade and reorganize dense networks. However, as T cells do not produce these enzymes, their migration to tumor sites can be significantly limited [91]. In this regard, including ECM degradation enzymes like heparanase [92] or inhibiting MMP proteins [93] has been shown to enhance CAR-T cell trafficking by facilitating their movement through the dense tumor matrix. However, MMPs comprise several members, each targeting distinct ECM substrates and playing unique roles in tumor progression and cell-cell interaction [89]. In addition, heparanase has functions beyond ECM degradation, as it is involved in various processes, such as regulating cancer stem cells [94], modulating chromatin and cell signaling pathways [95], promoting angiogenesis, and driving inflammation [90]. Next-generation sequencing technologies, particularly single-cell RNA sequencing, have significantly advanced our ability to deconvolute the immune landscape by predicting immune cell phenotypes, molecular landscapes, activation states, and lineage trajectories at single-cell resolution. This high-dimensional, high-resolution data provides cellular and molecular insights into TME heterogeneity and plasticity. When integrated with spatial and temporal contexts, these datasets become important inputs for constructing robust and dynamic DTs of the TME, capable of simulating immune cell interactions, therapeutic responses, and disease evolution with patient-specific precision [96].

Similar to antigen-centric modeling, DTs will benefit from various data, such as DSP, multi-omics, and high-throughput imaging, to address the TME complexity at the multi-scale level. Importantly, computational approaches capable of integrating diverse data types are essential for establishing robust and verifiable CAR-DTs. However, this integration significantly increases the complexity and computational demands of the framework [97]. As a solution, a modular design can be employed, starting with smaller, well-defined compartments, such as modeling the interactions between CAR-T cells and immunosuppressive cell populations. Over time, additional components of the TME can be integrated block-by-block through bottom-up or top-down methods, allowing for a scalable and manageable development process while maintaining model accuracy and interpretability [39]. This integrative approach enables the mapping of both inter- and intra-tumoral diversity, revealing the evolving landscape of immune suppression, stromal interactions, and tumor antigen variability. By combining these data layers, DTs can provide a more holistic and predictive framework for simulating CAR-T cell behavior and treatment response within the unique microenvironment of each patient.

Overall, the TME presents significant challenges for DT modeling due to its intricate molecular signaling networks and highly complex cellular organization. Therefore, it is crucial to consider the key actors of the TME when designing DTs to optimize CAR-T cell therapy, ensuring that the effects of the negative pressures exerted by the TME are minimized and therapeutic outcomes are maximized.

### 3.3. CAR-T Migration and Infiltration

A critical determinant of CAR-T therapy efficacy is the ability of T cells to effectively infiltrate the tumor niche. However, the success of CAR-T therapy in solid tumors is hindered by several trafficking and infiltration barriers.

The stroma is a non-malignant supportive tissue surrounding tumor cells and is organized in islets. It is enriched in blood vessels, endothelial cells, ECM, fibroblasts, and immune cells, which actively influence tumor growth, immune evasion, and therapy resistance [98]. For instance, the abnormal formation of tumor vessels, whether originating from tumor stem cells or endothelial precursor cells, restricts T-cell infiltration and impairs their anti-tumoral activity. Furthermore, tumor endothelial cells can directly kill T cells by increasing FasL expression while sparing Tregs, thereby further hindering effective immune responses [99]. In addition, CAR-T cell therapy can exacerbate endothelial dysfunction, leading to heightened macrophage inflammation, CAR-T cell toxicity, and CRS in vitro [100]. Interestingly, targeting vascular molecules with blocking antibodies [101] or CAR-T-targeted endothelial markers [102] has shown significant improvement in anti-tumoral activity, suggesting that modulating the tumor vasculature can enhance immune cell infiltration and function.

Angiogenic factors, such as VEGF, promote the formation of new blood vessels that supply oxygen and nutrients to tumors by stimulating the proliferation and migration of endothelial cells [103]. Interestingly, targeting VEGF receptors, such as VEGFR-1, VEGFR-2, and VEGFR-3, has emerged as a therapeutic strategy to disrupt the tumor vasculature and enhance CAR-T cell infiltration and function [104]. Despite promising results in targeting VEGF receptors in vivo, a Phase I/II trial using anti-VEGFR-2 was terminated due to the lack of a significant therapeutic response (NCT01218867). Lanitis et al. proposed a mechanism of failure, demonstrating that the limited efficacy of anti-VEGFR2 CAR-T cells in targeting tumors was due to the upregulation of VEGF-A, which outcompetes CAR-T cells and prevents them from effectively binding to VEGFR-2. This competition impairs CAR-T cell adhesion and their ability to destroy tumor cells in vitro. Co-administration of CAR-T cells with a VEGF-A-blocking antibody enhances the anti-tumor function of CAR-T cells [105]. Similarly, co-injection of a VEGF-blocking agent enhances T-cell infiltration into glioblastoma tissue, leading to delayed tumor growth in vivo compared to those infused with CAR-T cells alone [106].

CAR-T cells follow a multi-step process to reach tumor sites, which involves homing, extravasation, and migration through the tumor stroma. The initial homing process through blood vessels is mediated by chemokines, adhesion molecules, and cytokines [107]. However, solid tumors often modulate these molecules, especially chemokines and adhesion proteins [108,109], which are essential for leukocyte-endothelial interactions [110]. In addition, chemokines also recruit various immune cells, contributing to an immunosuppressive environment that further exerts additional constraints on the migratory function of T cells. For instance, monocytes and macrophages express the chemokine receptors CCR2 and CCR5, Tregs are recruited via CCR4, and MDSCs via CXCR1, CXCR2, CXCR4, and CCR2 [111]. Studies have shown that chemokine receptors, such as CCR1, CCR2 [112], CXCR4 [113], and CXCR5 [114], added to CAR-T cells, improve their anti-tumoral activity by maximizing their migration toward tumor and surrounding stroma cells.

Once at the tumor site, after passing the blood-endothelial cell barrier, CAR-T cells must overcome immune- and non-immune-cell-mediated barriers to penetrate the tumor mass effectively. For instance, cancer-associated fibroblasts (CAFs) constitute an important cellular component of the stroma, as they secrete ECM [115]. Wang et al. showed that the adoptive transfer of CAR-T cells directed against the fibroblast activation protein (FAP), a protease expressed by CAFs, inhibited tumor growth by depleting FAP-positive stromal cells [116].

To address the limitation of T cell infiltration in solid tumors, tumor-on-chip platforms have gained significant traction as innovative micro-engineered systems to address CAR-T cell infiltration deficiency in solid tissues [117]. Maulana et al. developed a breast-on-chip, a 3D model of breast microenvironment that mimics the endothelial barriers. In their system, the authors evaluated CAR-T cell infusion into the tumor, tumor cell lysis, and cytokine release. They assessed the optimal chemotherapy dosage to control the secretion of CAR-T-cell-derived CRS mediators without compromising CAR-T cell efficacy [118]. Although some organ-on-chip systems do not perfectly mimic the complexity of tumor tissues, the platform can serve as a real-time experimental system for DTs, offering a non-invasive platform to test various treatment regimens or engineered CAR constructs and investigate the limitations of CAR-T cell infiltration into tumor tissue. Therefore, combining tumor-on-chip technology with DTs can bridge the gap between bench and bedside, providing an alternative to invasive procedures and a lack of resources [119].

These studies suggest that targeting the stroma offers promising strategies to enhance T cell infiltration. However, selecting the appropriate target is crucial as it directly impacts CAR-T cell function. Therefore, DTs that integrate real-time insights using data derived from tumor-on-chip and other 3D systems can simulate and predict CAR-T cell migration into tissues. These models have the potential to identify optimal strategies for enhancing CAR-T cell infiltration and improving the therapeutic efficacy against solid tumors.

### 3.4. Acute Toxicity Associated with CAR-T Therapy

Severe immune-related toxicities following CAR-T cell infusion can emerge within the first week of treatment. The most prominent acute toxicities include CRS and immune effector cell-associated neurotoxicity syndrome (ICANS) [120].

CRS represents a significant side effect mediated by excessive cytokine production (e.g., IL-1, IL-6, IL-8, IL-10, MCP1, and IFN-γ), leading to systemic inflammation, fever, hypotension, and multi-organ dysfunction in severe cases [120]. CRS is classified based on severity using criteria such as the American Society for Transplantation and Cellular Therapy (ASTCT) grading system. Grade 1 (Mild) is characterized by fever (≥38 °C) without hypotension or hypoxia and often requires management of symptoms such as nausea, fatigue, and headache. Grade 2 (Moderate) includes fever with hypotension that responds to fluids or low-dose vasopressors and/or mild hypoxia requiring low-flow oxygen. Grade 3 (Prolonged) is characterized by significant hypotension requiring high-dose vasopressors hypoxia necessitating high-flow oxygen and is typically managed with IL-6 blockade and corticosteroids. Grade 4 (Life-Threatening) is characterized by refractory hypotension, respiratory failure, or critical organ dysfunction, requiring intensive care and aggressive immunosuppression [121]. Clinical parameters such as body temperature, respiration, blood pressure, and oxygen saturation, which are routinely monitored in patients undergoing CAR T cell therapy, provide immediate indicators of CRS [122]. These real-time physiological measurements can be directly fed into the DT framework to simulate patient-specific inflammatory trajectories and anticipate critical intervention points. In parallel, laboratory biomarkers such as C-reactive protein, ferritin, and liver function enzymes, which increase with CRS grade, serve as surrogate indicators of systemic inflammation and organ stress [120]. Tracking these values and their correlation with CAR-T cell dosing over time will enable DT to model the kinetics of toxicity and patient response, thereby supporting more informed clinical decisions regarding immunosuppression or dose modulation.

Mechanistically, cytokine release by CAR-T cells is crucial for their effector functions; however, the resulting cytokine response also stimulates monocytes and macrophages [123], which, in turn, produce additional pro-inflammatory cytokines, further amplifying the inflammatory response. Importantly, there is heterogeneity in the kinetics of CRS development based on co-stimulatory receptors. For example, CAR-T cells with a 4-1BB co-stimulatory domain tend to induce a slower, more controlled CRS response [124], while those with CD28 co-stimulation are associated with a more rapid and severe onset of CRS [125]. This variability demonstrates the complexity of the CRS response, which is influenced by the immune cell response, CAR-T construct, and proliferation dynamics associated with each co-stimulatory molecule.

ICANS, on the other hand, is a neurological complication that can occur within the first week in conjunction with CRS or independently and sometimes appears as an isolated complication [126]. The onset and severity of ICANS can vary widely among patients, with some experiencing mild symptoms that resolve quickly, while others may face significant neurological impairments requiring intensive management. Similar to CRS, ASTCT grades ICANS based on the severity of symptoms, ranging from Grade 1 (mild) with transient confusion and headache to Grade 4 (life-threatening), which includes symptoms such as seizures, delirium, and potentially irreversible cerebral edema [121]. These grades help guide clinical decision-making, including the use of corticosteroids and other immunosuppressive agents to manage and mitigate neurological toxicity. The development of ICANS is primarily driven by high infiltration of molecular and cellular components that break the blood-brain barrier (BBB), leading to central nervous system (CNS) inflammation. ICANS is associated with several secretory factors, including IFN-γ, IL-10, and granzyme B, which are found in the CNS and plasma [126]. Notably, factors linked to myeloid activation, such as GM-CSF, MCP-1, IP-10, IL-6, and IL-8, play prominent roles in the inflammatory response [127]. Interestingly, inhibition of GM-CSF, either through blocking antibodies or genetic knockout, reduces both CRS and ICANS in vivo while enhancing the efficacy of CD19-targeted CAR-T therapy [128]. Multiplex cytokine profiling, measuring levels of IL-6, IFN-γ, TNF-α, and other key cytokines, provides a dynamic snapshot of immune activation and toxicity grade [126]. These data streams are essential for refining DT simulations of immune-tumor interactions, CAR T cell activation thresholds, and cytokine-driven feedback loops. By integrating this information, DTs can help forecast CRS and ICANS severity, treatment efficacy, and the impact of interventions, such as IL-6R blockade. Complementing these systemic markers, single-cell RNA sequencing could also provide granular insights into the mechanisms of inflammation associated with immune cell infiltration and lineage trajectories at single-cell resolution. This technology allows DTs to simulate inflammation breakpoints, immune exhaustion, or clonal expansion with high fidelity.

CAR-T cell activation also drives the activation of resident brain macrophages, known as microglia, which interact with other infiltrating myeloid cells from the periphery to exacerbate CNS inflammation and neuronal damage [126,127]. Myeloid cell infiltration has been linked to higher grades of ICANS [129]. This infiltration promotes inflammation, which compromises the integrity of the BBB by damaging endothelial cells [130,131]. This disruption increases vascular permeability, allowing excessive immune cell infiltration and intensifying neuroinflammation. Consequently, the severity of ICANS is determined by the extent of BBB disruption, cytokine levels, and immune activation.

In conclusion, DTs should be designed to capture the critical tipping points that cause toxicity after CAR-T cell infusion. These models, which focus on addressing acute toxicities, should also optimize strategies to sustain the therapeutic inflammatory effects of CAR-T cells while reducing toxicity to the lowest level possible.

### 3.5. CAR-T Manufacturing Complexity

DTs can streamline CAR-T cell manufacturing by modeling each step of the complex production process, from leukapheresis to reinfusion [132,133]. By simulating T-cell selection, activation, and expansion, DTs enable real-time monitoring, predictive adjustments, and optimization of quality control measures. This approach enhances efficiency, reduces variability, and ensures compliance with manufacturing standards, ultimately improving patient outcomes.

CAR-T cell production is a time-consuming process that involves multiple sequential steps, including leukapheresis, T-cell isolation, genetic modification, ex vivo expansion, formulation, and final reinfusion into patients. Because each CAR-T cell is unique, the manufacturing process requires rigorous testing to verify the proper CAR expression, functionality, and potency. Due to the multiple steps involved, manufacturing is a labor-intensive process that requires strict quality control, compliance with current good manufacturing practices (cGMP), and specialized infrastructure to ensure the efficacy and safety of the final product [134]. Overall, the entire CAR-T cell production process can take up to four weeks before the final product reaches the patient. However, this timeline may vary depending on several factors, including manufacturing protocols, quality control assessments, logistical challenges, and patient-specific considerations [135].

During leukapheresis, the success of therapy largely depends on the selection of specific T-cell subsets, as different subsets exhibit distinct properties that influence persistence, efficacy, and overall therapeutic outcomes. T-cell populations primarily consist of CD4⁺ helper T cells and CD8⁺ cytotoxic T cells, including various subsets. Broadly, the main T cell subsets used for CAR-T cell therapy are naïve T cells (Tn), stem cell memory T cells (Tscm), central memory T cells (Tcm), effector memory T cells (Tem), effector T cells (Teff), and T cells. Each is presented in different proportions and exhibits unique functional characteristics (Table 1). These cells are then activated to prime T cells for genetic modification and their subsequent expansion. While activation using soluble or bead-bound anti-CD3/CD28 antibodies is widely adopted [136], alternative strategies using autologous or artificial antigen-presenting cells have also been employed to stimulate T cells [137,138]. Because prolonged signaling can lead to exhaustion, some protocols even bypass activation entirely, demonstrating successful CAR-T cell generation within 24 h [139]. Ferdous et al. developed a DT environment that simulates CAR-T cell interactions with artificial antigen-presenting beads in a two-dimensional setting, utilizing a machine learning algorithm to optimize dosing strategies for activation and expansion. The model integrates diverse inputs gathered from the literature, including cell counts, cell phenotypes, and imaging data, to evaluate which data types yield the most accurate predictions. While the model is initially a digital model, the authors designed the framework to evolve into a fully realized DT system through real-time experimental calibration (further discussed in Section 4.3) [140].

Once activated, T cells undergo genetic modification to express the CAR construct, typically through lentiviral or retroviral transduction methods. However, non-viral approaches, such as transposon-based systems and CRISPR/Cas, are also being explored as alternative gene delivery methods to lower costs and minimize the risk of off-target genomic insertions [150,151]. All approved FDA cell therapies are typically based on lentiviral or retroviral vectors for gene integration. The CAR structure is composed of several functional domains: an extracellular antigen-binding domain (e.g., anti-CD19 or anti-BCMA), a spacer or hinge region that provides flexibility (e.g., derived from IgG1 or CD8α), a transmembrane domain (e.g., CD28 or CD8α), a co-stimulatory domain to enhance T cell activation and persistence (e.g., CD28 or 4-1BB), and a signaling domain, most commonly CD3ζ, responsible for initiating T cell activation.

Following genetic modification, CAR-T cells should be expanded ex vivo under controlled conditions using cytokine supplementation (e.g., IL-2, IL-7, and IL-15) to achieve a clinically relevant dose. This step is critical for generating a sufficient number of potent and functional CAR-T cells while maintaining the memory phenotype and persistence.

Before infusion, the CAR-T product undergoes quality control assessments, including sterility testing, transduction efficiency, viability, purity, and potency assays, to confirm its functional tumor-killing capacity [152]. Once approved, the CAR-T cells are either cryopreserved or administered fresh into the patient following lymphodepletion by chemotherapy (e.g., cyclophosphamide and fludarabine) to enhance engraftment and persistence [153].

Post-infusion, patients will undergo intensive monitoring for potential toxicities, as discussed above for CRS and ICANS, as well as long-term assessments of patient response to therapy and long-term persistence of CAR-T cells. Despite its success, CAR-T cell therapy faces manufacturing challenges, including production time, variability in manufacturing protocols, heterogeneity in patient-derived T-cell quality, and high costs [154].

Therefore, DTs should implement automated manufacturing systems that not only optimize production efficiency but also enable the personalization of CAR-T therapies. The virtual replica should facilitate real-time adjustments in the manufacturing process to ensure maximum therapeutic efficacy. A model proposed by Shoshi et al. designed a modular DT framework to address the high costs and complexity of CAR-T cell manufacturing. Their hierarchical system integrates automation, digitization, and real-time monitoring to enhance process efficiency, traceability, and regulatory compliance [133] (the model is further discussed in Section 4.3).

In summary, multiple DT models can be developed at various stages of the therapeutic pipeline, ranging from molecular interactions to patient-specific therapy simulations and post-infusion monitoring. These layered models allow for comprehensive end-to-end optimization, creating an integrated infrastructure that enhances the understanding of CAR-T cell therapy, safety, and efficacy.

## 4. Case Studies of DT for CAR T Cells

To date, several DTs have been developed to address various aspects of CAR-T cell therapy, including CAR-T cell kinetics, CRS evaluation, and automated workflows for CAR-T cell manufacturing.

### 4.1. Optimizing the Cell for CAR-T Selection

Joslyn et al. developed a quantitative systems pharmacology model to characterize the kinetics of T cells following infusion [155]. The model incorporates important processes, including lymphodepletion, proliferation, trafficking, differentiation, and apoptosis across the periphery and tumor tissue compartments. It specifically focuses on T cell subsets, including Tscm, Tcm, Tem, and Teff. Individual DTs were generated for a cohort of 10 patients to simulate treatment responses across three dosing levels: low, medium, and high. Patient-specific DTs were created to replicate the T cell kinetics observed in a clinical trial targeting the E7 neoantigen in metastatic HPV-associated cancers. Using this strategy of individualized models, the authors were able to evaluate variability among patients and across different dose groups. Interestingly, the analysis shows Tem proliferation and Tem conversion remain consistent across patients and dose groups, indicating that they do not contribute to interpatient variability in the kinetic model. In contrast, the proliferation rate of Tscm cells varies significantly within and across the dose groups, influencing the T cell counts and driving variability among patients. Therefore, the model identified Tscm as critical for the expansion and persistence of engineered T cells, highlighting Tscm enrichment as a strategy to improve therapeutic outcomes and reduce the dose requirements. Broader applicability was confirmed by predicting T cell kinetics in patients with pancreatic cancer treated with KRAS G12D-targeted therapy, which showed the relevance of Tscm cells in CAR therapy [155].

### 4.2. Clinical Trials

Li et al. developed a DT to define the distribution of CRS among lymphoma patients treated with CAR-T cell therapy, modeling treatment safety and efficacy outcomes [156]. The development process adhered to the Knowledge Discovery in Databases (KDD) framework, containing several systematic steps: selecting and integrating data from multiple sources, preprocessing to address inconsistencies, transforming data for meaningful analysis, applying data mining techniques to identify CRS-related patterns, evaluating these patterns for relevance, and representing the knowledge gained in actionable formats. Baseline data from 5473 patients and individual records from 138 patients were analyzed. After applying specific inclusion and exclusion criteria based on age, diagnosis, treatment history, CRS grade, and performance status, a cohort of 392 patients was selected to construct the DTs of the population. These patients were grouped into eight cohorts and assigned CRS grades ranging from 1 to 4. The CRS-DT demonstrated consistency across cohorts, with high-grade CRS (Grades 3 and 4) being rare (7% of cases) and median-grade CRS (Grade 2) being the most prevalent (44% of cases). Despite the relatively small sample size, the data source, whether from individual records or grouped specimens, did not impact the conclusions. This study highlights the importance of developing CAR-T cell therapy strategies that focus on reducing the occurrence of high-grade CRS and significantly lowering the prevalence of moderate-grade CRS. Additionally, this study demonstrated the feasibility of using retrospective and prospective analyses to reflect CRS distributions across multiple cohorts. In conclusion, a DT trial arm based on individual DTs can be built to evaluate a comparable CRS-by-grade distribution pattern and serve as a virtual substitute cohort for real-world clinical trials, facilitating the interpretation of trials while maintaining rigorous scientific standards [156].

### 4.3. Manufacturing

Two studies addressed CAR-T cell manufacturing, with the first focusing on enhancing CAR-T cell activation and the second examining the entire manufacturing process end-to-end.

In the first model, Ferdous et al. built a DT environment to simulate optimal dosing for CAR T-cell activation and expansion [140]. Using artificial antigen-presenting beads (aAPCs) to activate naïve T-cells as a 2D simulation, the study models how reinforcement learning (RL), a subset of machine learning, can control the timing and dosing of aAPC exposure to maximize the yield of effector T-cells while minimizing exhaustion. As input, the RL algorithm receives three types: (i) cell counts and cell types, (ii) 2D imaging environment, and (iii) a combination of both. In addition, the framework accommodates a range of simulated cell phenotypes, including exhaustion rate, activation probability, exhaustion, regeneration dynamics, and division mode (e.g., symmetric and asymmetric division), to reflect CAR-T cell biological transitions. These inputs were processed to inform the agent’s control strategy, which is refined over multiple episodes based on the reward outcomes, allowing the model to progressively learn and optimize its decisions. Notably, the authors developed a modular framework capable of real-time data updates and outlined the key steps to advance the model toward a fully realized digital twin system. These steps include transitioning from 2D to 3D simulations, integrating continuous data streams from the physical system, and refining the model parameters using experimental data.

In the second study, Shoshi et al. developed DT framework designed for CAR-T cell production [133]. Their work addresses the high development and manufacturing costs driven by stringent quality assurance and regulatory compliance requirements, including FDA guidelines and current good manufacturing practices (cGMP). Given the complexity of the production chain, rigorous monitoring by highly trained personnel is essential to ensure comprehensive process tracking and traceability. These challenges highlight the critical need for advanced monitoring and control mechanisms at each stage of the manufacturing process. To address these challenges, the authors integrated automation, digitization, and real-time monitoring into their DT framework. Automation minimizes human intervention to enhance workflow while reallocating skilled personnel to critical roles. Digitization aims to translate physical production processes into digital replicas through real-time data synchronization. Real-time monitoring ensures seamless oversight across all automation levels, from manual to fully automated systems. The framework was structured into four hierarchical levels: (i) Processes, representing key production stages such as extraction, T cell manipulation, and CAR engineering; (ii) Tasks, modular components that describe the detailed steps based on operations performed by humans, machines, or software within a process; (iii) Skills, specifying how tasks should be executed by either humans or machines; and (iv) Microservices, smaller execution units linked to human or machine actions, ensuring traceability and identifiability. The authors designed the framework as modular components that can be individually updated without disrupting the overall system.

This study provides a flexible workflow for supporting the constraints of CAR-T cell manufacturing and paves the way for more cost-effective, scalable, and precise production processes [133].

## 5. Limitations and Challenges of DTs

### 5.1. Data Availability and Quality

Generating a high-fidelity CAR-DT will require comprehensive multi-omics [157], single-cell [158], and longitudinal clinical data during the whole process of CAR-T cell therapy, which may not always be readily available due to technical and logistical limitations. For instance, Li et al. (Section 4.2) started with a cohort of 5473 patients but employed 393 cases to create DTs, suggesting that extensive data preprocessing and filtering may be required to guarantee that the model is based on relevant, high-quality data. This reduction in cohort size emphasizes the difficulties in collecting sufficient precise data and the necessity of strict selection standards to find the most instructive datasets [156]. Moreover, the process may become even more complex and expensive if such data must be gathered over long periods [159,160]. The accuracy and usability of CAR-DT in clinical settings may be impacted by the gaps in the model’s representation of patient-specific tumor dynamics, immune responses, and treatment outcomes.

A key challenge specific to CAR-T therapy is that T cells are derived from the patient’s immune system, which makes CAR-T cell responses personalized, unlike other therapies. Therefore, an effective CAR-DT may require the reconstruction of the pre-infusion tumor-immune ecosystem to establish a baseline before therapy. This involves mapping the patient’s unique immune profile, tumor antigen expression, and interactions within the TME to identify potential barriers to treatment efficacy. Then, what started as an individual model can be expanded to a larger group to build a virtual clinical trial [56]. However, the heterogeneity of data sources across institutions introduces inconsistencies in data collection methods, making it challenging to develop standardized, generalizable models.

Once CAR-T cells are infused, real-time data integration becomes essential for tracking post-infusion responses, capturing T-cell expansion, exhaustion, antigen escape, and dynamic changes in the TME. However, real-time monitoring at this level requires advanced data collection technologies, such as continuous biomarker tracking, liquid biopsies, and imaging modalities, which can be a constraint for patients. To ensure the successful application of CAR-DTs across different clinical settings, it will be vital to establish a standardized framework for integrating multiple DTs developed at various cancer centers. This framework would ensure harmonization, consistency, reliability, and scalability in real-world clinical use [161]. Such standardization will also support broader collaboration and data sharing, accelerating the development of personalized and adaptive CAR-T therapies.

### 5.2. Model Development and Complexity

Computational approaches are often limited in scope and are highly specific to the study context. Due to spatial and temporal heterogeneity, it is particularly challenging to simultaneously model tumor progression, immune evasion strategies, and patient-specific treatment responses into a single model [162].

Because computational approaches rely on available data, oversimplification of complex biological systems can significantly impact their predictive power. If the data used to inform the model are incomplete, noisy, or unrepresentative, the model’s predictions may be unreliable. Choosing certain parameters over others or making simplifying assumptions about biological interactions can bias the results and impede the model’s real-world applicability [163].

Additional uncertainty arising from patient variability, tumor evolution, and unpredictable immune responses can be challenging to assess, introducing significant noise that current models may struggle to adequately account for. Therefore, these models may struggle to fully represent the uncertainties present in real-world biological systems. As a result, computational models can be subjected to bias due to the assumptions made during model design or inherent limitations in the available data.

Lastly, scaling these models to address the vast complexity of large biological systems, including molecular, cellular, and histological scales, remains a considerable challenge. As complexity increases, so do the computational demands, which can exceed the capabilities of current modeling tools and infrastructures. Some approaches have been proposed to build it gradually using bottom-up or top-down methods [39]. The bottom-up method builds models based on fundamental molecular and cellular interactions. This strategy integrates detailed mechanistic insights, such as receptor-ligand binding dynamics, cytokine response, and immune cell activation, to create granular representations of biological processes [164]. However, bottom-up models can become computationally expensive and may struggle to capture emergent system-level behaviors due to the number of parameters involved. Conversely, the top-down method begins with large-scale clinical data and identifies treatment responses without explicitly modeling every molecular interaction [165,166]. While top-down models are often easier to use, they may lack mechanistic interpretability and risk oversimplifying critical biological interactions.

Addressing these challenges requires multi-scale, longitudinal patient data, real-time monitoring technologies, and advanced computational algorithms to refine predictive accuracy. It is important to prioritize the most critical components that influence CAR-T cell therapy during the design of DTs.

### 5.3. Computational and Technological Barriers

The clinical implementation of these CAR-DTs may face several technical and standardization challenges.

A significant difficulty is still reaching a balance between DTs’ real-time adaptability and computational efficiency. Integrating multi-omics, imaging, clinical records, and treatment responses will require harmonizing diverse datasets into a unified model. Differences in format, resolution, and acquisition methods create challenges in standardization and interoperability. To address this issue, advanced algorithms must normalize the data, reduce biases, and ensure comparability across modalities [167]. Model fusion may become increasingly challenging as the number of CAR-DT models increases, as different models may concentrate on specific facets of CAR-T cell biology, requiring advanced computational methods to fill these gaps [168]. This variability can lead to inaccurate predictions, reduced reproducibility, and diminished clinical reliability across research centers and institutions. To address these issues, building individualized patient-specific DTs for personalized treatment could provide more accurate predictions. Integrating these personalized models into a broader framework could generate virtual cohorts, enabling more robust simulations and enhancing the generalizability of the findings. This approach would allow for better-informed clinical decisions and the ability to evaluate treatment strategies at the population level while still maintaining the specificity required for individual patients [34].

Because the development of DTs in oncology is still in its early stages, standardized guidelines for defining data collection protocols, model validation criteria, model fusion, and clinical integration strategies have yet to be established [169,170]. Therefore, establishing harmonized regulatory frameworks, validation pipelines, and interoperability standards is essential to ensure that DTs can be effectively implemented and trusted in clinical decision-making.

Running complex multi-scale simulations will require significant computational power, making high-performance computing (HPC) essential. However, not all clinical settings may be able to meet these objectives, particularly in institutions with limited resources. The cost of HPC infrastructure will initially be expensive for healthcare providers, creating disparities in healthcare and contributing to global inequalities [9]. Further barriers to broad adoption include the expense of maintaining and updating technology and the requirement for training specialist staff to oversee complex simulations.

Overcoming these barriers will require advancements in computational efficiency, regulatory alignment, and standardized methodologies. By addressing these challenges, DTs could become a scalable and clinically viable tool for enhancing patient outcomes and customizing CAR-T cell therapy.

## 6. Conclusions

CAR-T cell therapy has transformed immunotherapy strategies by harnessing the patient’s own T cells to recognize and eliminate tumors. However, challenges such as antigen escape, TME, therapy resistance, and patient-to-patient variability underscore the need for more advanced predictive tools. DTs offer an innovative solution by creating a virtual replica of a patient’s immune system and TME, leading to in silico testing of different CAR construct signaling fitness, tumor antigen targets, and combination therapies. By continuously integrating real-time patient data, DTs can evolve into dynamic, precision-driven models that optimize CAR-T cell therapy strategies. These models have the potential to increase long-term survival by managing therapy-related risks and improving the treatment efficacy. However, for DTs to be clinically transformative, they must first accurately replicate complex biological systems, undergo rigorous validation, and meet regulatory standards. As these models progress, their seamless integration into clinical workflows could redefine CAR-T cell therapy, paving the way for a new era of predictive, adaptive, and patient-centered cancer treatments.

## Figures and Tables

**Table 1:** Summary of pros and cons of T cell subsets in CAR-T cell therapy.

T Cell Subsets	Pros	Cons	References
Naive T cells (Tn)	- High proliferative potential and longevity - Capable of differentiating into various T-cell subsets - Associated with less toxicity	- Limited persistence - Require priming and activation - Limited immediate cytotoxic function	[141,142]
Stem Cell Memory T Cells (Tscm)	- Self-renewal capability and high lifespan - Fast response upon antigen stimulation - Can differentiate into multiple T-cell lineages - Associated with less toxicity	- Less abundant in circulation - Variable activation conditions	[142,143,144]
Central Memory T Cells (Tcm)	- Long-term persistence and self-renewal - High cytotoxicity potential - Enhanced homing to lymphoid tissues	- Delayed immediate cytotoxic response - May require additional activation	[145,146]
Effector Memory T Cells (Tem)	- Rapid effector function and immediate cytotoxicity - Effective tumor infiltration	- Shorter persistence compared to Tcm - Reduced self-renewal capacity	[147,148]
Effector T Cells (Teff)	- Strong immediate anti-tumor activity - High cytokine production and cytotoxic function	- Limited persistence - lack of self-renewal capacity - Prone to exhaustion	[148,149]
